# Increased Na^+^/Ca^2+^ Exchanger Activity Promotes Resistance to Excitotoxicity in Cortical Neurons of the Ground Squirrel (a Hibernator)

**DOI:** 10.1371/journal.pone.0113594

**Published:** 2014-11-21

**Authors:** Juan-Juan Zhao, Shan Gao, Jun-Zhan Jing, Ming-Yue Zhu, Chen Zhou, Zhen Chai

**Affiliations:** State Key Laboratory of Biomembrane and Membrane Biotechnology, College of Life Sciences, Peking University, Beijing, China; National University of Singapore, Singapore

## Abstract

Ground squirrel, a hibernating mammalian species, is more resistant to ischemic brain stress than rat. Gaining insight into the adaptive mechanisms of ground squirrels may help us design treatment strategies to reduce brain damage in patients suffering ischemic stroke. To understand the anti-stress mechanisms in ground squirrel neurons, we studied glutamate toxicity in primary cultured neurons of the Daurian ground squirrel (*Spermophilus dauricus*). At the neuronal level, for the first time, we found that ground squirrel was more resistant to glutamate excitotoxicity than rat. Mechanistically, ground squirrel neurons displayed a similar calcium influx to the rat neurons in response to glutamate or N-methyl-D-aspartate (NMDA) perfusion. However, the rate of calcium removal in ground squirrel neurons was markedly faster than in rat neurons. This allows ground squirrel neurons to maintain lower level of intracellular calcium concentration ([Ca^2+^]_i_) upon glutamate insult. Moreover, we found that Na^+^/Ca^2+^ exchanger (NCX) activity was higher in ground squirrel neurons than in rat neurons. We also proved that overexpression of ground squirrel NCX2, rather than NCX1 or NCX3, in rat neurons promoted neuron survival against glutamate toxicity. Taken together, our results indicate that ground squirrel neurons are better at maintaining calcium homeostasis than rat neurons and this is likely achieved through the activity of ground squirrel NCX2. Our findings not only reveal an adaptive mechanism of mammalian hibernators at the cellular level, but also suggest that NCX2 of ground squirrel may have therapeutic value for suppressing brain ischemic damage.

## Introduction

Hibernation is a physiological process characterized by inactivity with a low metabolism and body temperature (the torpor state). It is regularly interrupted by brief periods of arousal when the metabolism and body temperature return to normal. During hibernation, blood flow to the brain is dramatically reduced and can be as low as one tenth of the normal level [1]. Hibernators can endure tremendous decrease in oxygen-glucose supply without neurological injury, as observed *in vitro* and *in vivo* [1]–[5]. Therefore, hibernators can be served as an ideal model for studying tolerance to brain ischemia and ischemia-related injuries. Interruption of the blood flow to the brain during ischemic stroke severely compromises the energy supply to the brain, which further disrupts ionic gradients across plasma membrane. This leads to neuronal depolarization, enhanced excitatory neurotransmitter release and reduced transmitter reuptake. Excessive extracellular accumulation of glutamate overactivates glutamate receptors, elicits calcium overload, and can ultimately lead to neuronal death [6]–[9].

In neurons, calcium functions as a signal in a variety of processes, including transmitter release, synaptic plasticity and gene transcription. At rest, the global intracellular calcium concentration ([Ca^2+^]_i_) of most neurons is approximately 50∼100 nM [10], which, after excitation, rises transiently to a level that is dozens of times higher [11]. Prolonged exposure to a high concentration of calcium overactivates endonucleases, proteases, lipases and phosphatases, which leads to lethal downstream reactions driven by oxidative stress or mitochondrial dysfunction [12]. The balance between calcium influx to the cytosol and calcium removal from the cytosol maintains calcium homeostasis. Calcium influx through the channels on the plasma membrane and calcium release from intracellular calcium stores are the two sources of [Ca^2+^]_i_ elevation. Once neurons are activated, calcium from the extracellular fluid enters the cell through multiple ways, including voltage-gated calcium channels, ionotropic glutamate receptors (such as N-methyl-D-aspartate (NMDA) receptors and calcium-permeable α-amino-3-hydroxy-5-methyl-4-isoxazolepropionic acid (AMPA) receptors), nicotinic acetylcholine receptors [13], transient receptor potential channels [14] and store-operated channels [15], [16]. In addition, when ryanodine receptors or inositol trisphosphate receptors are activated, calcium is released from the endoplasmic reticulum (ER). After activation, the calcium concentration returns to resting level by pumping calcium out of the cell and/or back into internal stores via the Na^+^/Ca^2+^ exchanger (NCX), the plasma membrane calcium ATPase (PMCA) and the sarco/endoplasmic reticulum calcium ATPase (SERCA), respectively. Meanwhile, mitochondria can buffer calcium by transiently taking up calcium through the uniporter and slowly releasing it back into the cytosol through the mitochondrial NCX [10].

To date, our understanding of the adaptive mechanisms of mammalian hibernators to brain ischemic injury is limited. Studies in injury models have been performed mostly on brain slices, and the tolerance at the cellular level was undetermined. Moreover, most studies have focused on injury tolerance during the hibernating state with few concerning the euthermic state [2], [17]. In addition, the role of excitotoxicity-induced calcium overload (a central event in ischemic injury) has not been well investigated in hibernators. In the present study, we determined the tolerance against glutamate toxicity of Daurian ground squirrels (which are hibernators) in primary cultured cortical neurons and investigated the adaptive mechanisms of ground squirrels at the neuronal level.

## Materials and Methods

### Animals and ethics statement

Daurian Ground squirrels [(*Spermophilus dauricus* (Brandt 1843)] and Sprague-Dawley rats [*Rattus norvegicus* (Berkenhout 1769)] were manipulated in accordance with the strict guidelines of the 7^th^ edition of the Guide for Care and Use of Laboratory Animals published by the US National Institutes of Health. Animal trapping and experiments were approved by the Institutional Animal Care and Use Committee of Peking University (Permit Numbers: lsc-wangsq-1 and lac-tianyl-2). The ground squirrel used in the present study, *Spermophilus dauricus*, is a species widely distributed in China, Mongolia and Russia. In approximately mid-May, pregnant ground squirrels were caught in Zhangbei County (41.2°N, 114.8°E; Hebei province, China) and fed cabbages and rodent chow, following a 7-day quarantine period.

### Primary culture of cortical neurons and glutamate exposure

Primary cortical neurons were prepared from 18-day-old Sprague-Dawley rat embryos (Vital River Laboratory Animal Technology Co., Ltd., Beijing, China) and postnatal day 1 ground squirrels as previously described [18]. To minimize suffering, the fetal rats and neonatal ground squirrels were rapidly decapitated before cortices were removed. The dissociated cerebral cortices were then digested with 0.2% trypsin (Gibco, Gaithersburg, MD, USA) at 37°C for 2 minutes, and the digestion was terminated with 0.1% trypsin inhibitor (Sigma, St. Louis, MO, USA). The dispersed cells were collected and plated on poly-D-lysine pre-coated coverslips or 24-well plates at a density of 0.5–0.8×10^5^ cells/cm^2^ in neurobasal medium (Gibco) supplemented with 2% B27 (Gibco), 2 mM glutamine (Sigma), 50 units/ml penicillin and 50 µg/ml streptomycin (Gibco). Forty-eight hour later, 10 µM cytosine β-D-arabinofuranoside (Sigma) was added, followed by changing the culture medium after 24 hours. Neurons were kept in a 5% CO_2_, humidified incubator at 37°C for 8–10 days before experiments, and half of the culture medium was replaced every 3 days. Immunofluorescence analysis showed that astrocyte contamination was less than 3% in rat neurons [18], the purity of ground squirrel neurons was more than 98%. After culturing for 9 days *in vitro*, the primary neurons were exposed to 200 µM L-glutamate (Sigma), and the neuronal viability was determined 3 hours or 24 hours later.

### Cell viability by MTT, ATP luminescence and LDH release assays

For the MTT assay, neuronal metabolic activity was determined by measuring the conversion of 3-(4,5-dimethylthiazol-2-yl)-2,5-diphenyltetrazolium bromide (MTT) into a purple formazan product. Briefly, MTT (sigma) was dissolved in neuronal culture medium at a concentration of 0.5 mg/ml and 300 µl was added to each well. After 3 hours of incubation at 37°C, the culture medium was removed, and 300 µl/well of DMSO (Merck, Whitehouse Station, NJ, USA) was added to dissolve the formazan crystal. The absorbance was read at a wavelength of 570 nm on a microplate reader (BIO-RAD Model 680, Hercules, CA, USA).

For the ATP assay, ATP levels in neurons were determined using the CellTiter-Glo luminescent cell viability assay (Promega, Fitchburg, WI., USA) according to the manufacturer's instructions. The primary neurons were lysed for 2 min with a mixture of CellTiter-Glo reagent and neurobasal medium after exposure to glutamate for 24 hours, followed by 10 min incubation in the same mixture at room temperature to stabilize the luminescent signal. Wells containing CellTiter-Glo reagent and medium without cells were used as control for measuring the background luminescence. The overall luminescence was recorded in opaque-walled multiwell plates (Corning Inc., Corning, NY, USA) with a Thermo, Varioskan Flash microplate reader (Thermo Scientific, USA). The ATP level in glutamate treated neurons was expressed as a percentage of that in control neurons.

For the lactate dehydrogenase (LDH) release assay, media from cultured neurons were collected, and the LDH activity was measured using a commercial kit (CytoTox 96 Non-Radioactive Cytotoxicity Assay, Promega) in accordance with the manufacturer's instructions.

### Measurement of the reactive oxygen species

Dihydroethidium (DHE; Sigma) was used to measure the reactive oxygen species (ROS) levels in primary neurons of rat and ground squirrel. Neurons loaded with 10 µM DHE for 10 min at 37°C were imaged with an inverted microscope (20/NA 0.45, Olympus IX71,Tokyo, Japan) in artificial cerebrospinal fluid (ACSF; 141 mM NaCl, 2.5 mM KCl, 1.3 mM MgCl_2_, 1.25 mM NaH_2_PO_4_, 10 mM glucose, 2 mM CaCl_2_ and 10 mM HEPES; pH 7.4). Neurons treated with glutamate were loaded with DHE solution with 200 µM glutamate and recorded in ACSF supplemented with 200 µM glutamate.

### Measurement of mitochondrial membrane potential (Ψ_m_)

A ratiometric probe 5,5′,6,6′-tetrachloro-1,1′,3,3′ tetraethylbenzimidazolyl-carbocyanine iodide (JC-1, Biotium Inc., Hayward, CA, USA) was used to detect the mitochondrial membrane potential (Ψ_m_) in primary neurons. The monomeric green form and the aggregate red form of JC-1 have maximum emissions at wavelengths of 530 and 590 nm. Primary neurons loaded with JC-1 were kept in the incubator for 15 min before imaging with a confocal microscope (40/NA 1.0 water-immersion lens, Zeiss 710, Oberkochen, Germany). JC-1 was excited at 488 nm, and the red/green components of the emission fluorescence were spectrally separated. Neurons treated with glutamate were loaded with JC-1 solution supplemented with 200 µM glutamate, and then recorded in ACSF with 200 µM glutamate.

In transfection experiments, neurons were loaded with 100 nM tetramethylrhodamine ethyl ester (TMRE, Invitrogen, Carlsbad, CA, USA) for 10 min at 37°C to measure Ψ_m_.

### [Ca^2+^]_i_ measurement

The resting Ca^2+^ concentration was measured with the ratiometric calcium indicator fura-2 AM. The cultured neurons were rinsed with ACSF after the culture medium removal. Then, 5 µM fura-2 AM (Invitrogen, Carlsbad, CA, USA) was added for 10 min at 37°C avoiding light. Next, the cultures were placed on an inverted microscope (20/NA 0.45, Olympus IX71, Tokyo, Japan) after being rinsed twice with ACSF. The same process was performed for glutamate treated neurons, except for same concentration of glutamate (200 µM) added in all of the solutions contained. 3-amino-6-chloro-5-((4-chlorobenzyl)amino)-N-(((2,4-dimethylbenzyl)amino)iminomethyl)-pyrazinecarboxamide (CB-DMB, Sigma) was used to block NCXs; and thapsigargin (TG, Sigma) was used to block SERCA. All images were taken with the Cell∧R system (Olympus). The excitation light from a xenon lamp was filtered with a rotating wheel containing 340 nm and 380 nm filters (Semrock, Rochester, NY, USA). The emitted fluorescence was measured at a wavelength approximately 510 nm. All the images were analyzed with Xcellence software (Olympus). The [Ca^2+^]_i_ was calculated using the Eqn 1:
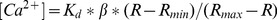
(1)where *R* = F_340_/F_380_; *K*_d_ is the dissociation constant of fura-2; *β* = F_380min_/F_380max_, and *R*_min_ and *R*_max_ are the minimal and maximal F_340_/F_380_. Following the method described by Sipido [19], the parameters in Eqn 1 were determined and shown in Eqn 2:
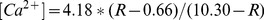
(2)To calculate the calcium removal rate, fluo-4 AM (Invitrogen), a calcium indicator with a faster rate of Ca^2+^ dissociation than fura-2, was used. After being rinsed twice with ACSF, the neurons on the culture coverslips were loaded with 10 µM fluo-4 AM for 10 min at 37°C and then placed on an inverted microscope. Upon glutamate perfusion, calcium transients were recorded with a laser scanning confocal microscope (Zeiss 710, 40 water-immersion lens/NA 1.0). All of the experiments were performed at 25°C. The acquired images were processed with IDL 7.0 software (Research Systems, Inc., Boulder, CO, USA). The calcium concentration was calculated according to the Eqn 3:
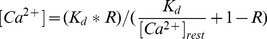
(3)in which *R* = F/F_0_; *K*_d_, the dissociation constant of fluo-4, is almost temperature-independent at physiological pH, and an approximate value of 1.1 µM was used in the formula [20]. The resting calcium concentration in primary neurons, [Ca^2+^]_rest_, was determined as 94 nM by using the calcium ratiometric indicator fura-2 AM. Rhod-2 AM (Invitrogen) was used as a calcium indicator to calculate the calcium removal rate in neurons 2 days after transfection with plasmids carrying *EGFP*. The *K*_d_ value for rhod-2 was 1.3 µM [21]. The decay phase of the [Ca^2+^]_i_ transient was fitted with a 1-phase exponential decay curve and the time constant (τ) was then obtained using the GraphPad Prism 5.0 software (GraphPad Software Inc., San Diego, CA, USA).

### Electrophysiology

Whole-cell NMDA current recordings were conducted on the primary neurons cultured for 9–10 days *in vitro* in ACSF. Patch electrodes (4–6 MΩ) were fabricated on a horizontal puller (Sutter, Instruments). The pipette solution contained the following components: 115 mM K-gluconate, 20 mM KCl, 1.5 mM MgCl_2_, 10 mM HEPES, 0.025 mM EGTA, 0.2 mM Li_4_-GTP, 2 mM Mg-ATP and 10 mM Na_2_-phosphocreatine. The pH was adjusted to 7.2–7.3 with KOH. NMDA currents were evoked with magnesium-free ACSF with 100 µM NMDA and 10 µM glycine at the holding potential of −70 mV. Whole-cell patch-clamp recordings were made with an EPC-10 amplifier (HEKA Electronics, Lambrecht, Germany) and digitized at 10 kHz with Patchmaster software (HEKA). Charge transfer density was calculated as the area under the curve (pA*s) normalized to cell capacitance (pF).

### NCX cloning, plasmid preparation, cell transfection and determination of cell viability

Total RNA was extracted from the cerebral cortical tissue of rats and ground squirrels (male, approximately 200 g) using TRIzol (Invitrogen) according to the manufacturer's instructions. The purified RNA was reverse transcribed into cDNA with oligo (dT) 15 primers. Rat and ground squirrel *NCXs* were cloned using the primers listed below (5′-3′; ground squirrel: GS): Rat-NCX1-F ATGCTTCGACTAAGTCTC; Rat-NCX1-R TTAGAAGCCTTTTATGTG; Rat-NCX2-F ATGGCTCCCTTGGCTTTG; Rat-NCX2-R CTAGAAGCCCCGAATGTG; Rat-NCX3-F TGTATGGCGTGGTTACGG; Rat-NCX3-R GCCCTGTGGAGGTCTTGT; GS-NCX1-F ATGCCTCGGTTAAGCCTC; GS-NCX1-R TTAGAAGCCTTTTATGTG; GS-NCX2-F ATGGCTCCCCTGGCTTTGGTG; GS-NCX2-R CCTAGAAACCCCGGATGTGGC; GS-NCX3-F ATGGCGTGGTTAAGGTTG; GS-NCX3-R GTGGATTTGTTGCTGTTG. The sequence data of rat *NCX*s are available on GenBank (*NCX1*-*NCX3*: accession numbers: NM_001270774, NM_078619, NM_078620; version numbers: NM_001270774.1, NM_078619.1, NM_078620.1). The primers for ground squirrel *NCXs* were designed according to the sequence data of squirrel on Ensembl (Ensembl version: ENSSTOG00000025770.1, ENSSTOG00000020165.1, ENSSTOG00000003907.2). PrimeSTAR Max DNA Polymerase (Takara Biotechnology (Dalian) Co., Ltd., Dalian, China) was used to amplify the cDNA template according to the following PCR protocol: 35 cycles of 98°C for 10 s, 55–60°C for 15 s and 72°C for 20 s. Nucleotide sequence encoding for 2A peptide from porcine teschovirus-1 (P2A; GCTACTAACTTCAGCCTGCTGAAGCAGGCTGGAGACGTGGAGGAGAACCCTGGACCT) were synthesized by Ruibio Tech (Beijing, China), and annealed before they were inserted into a modified version of pFUGW ([22]; courtesy of Prof. Zhang from Peking University) at the BsrGI/BamHI (restriction endonucleases from NEB, Beverly, MA, USA) sites; this construct was termed pFUGW-P2A when mentioned. NCX cDNAs were cloned into pFUGW-P2A at the NheI/BamHI sites. After 6 days *in vitro*, cortical neurons were transfected with lipofectamine 2000 (Invitrogen) for 12 hours.

Neuronal viability was determined by assessing cell and nucleus morphology after staining with 0.4 µg/ml Hoechst 33342 (Sigma) for 10 min at 37°C. All of the images were taken under a fluorescence microscope (Olympus IX71, 10/NA 0.30).

### Single-cell PCR analysis of ground squirrel NCX2 expression

Primary neurons were suspended in RNase-free PBS following digestion with trypsin-EDTA at 4–6 hours after removing the transfection medium (Gibco, Gaithersburg, MD, USA). Individual target neuron was sucked into a patch electrode (tip diameter ranging 15–20 µm) under a fluorescence microscope (Olympus IX71, 20/NA 0.45), and then transferred immediately to liquid nitrogen in a PCR tube (Axygen, Hangzhou, China). After incubating at 70°C for 90 s, oligo (dT) 15 primers were added for reverse transcription using SuperScript III (Invitrogen). PrimeSTAR Max DNA Polymerase (Takara Biotechnology (Dalian) Co., Ltd., Dalian, China) were added for target amplification. The expression level of ground squirrel *NCX2* was determined by PCR amplification with primers GS-NCX2 (F: 5′TCATCGCTGACCGCTTCA3′; R: 5′GCAGGCGTCCTCATAGTGC3′) according to the following protocol: 30 cycles of 98°C for 10 s, 59°C for 15 s and 72°C for 20 s. As an internal control, *beta-actin* expression was determined with primers (set 1, F1: 5′GAAATCGTGCGTGACATTA3′, R1: 5′ACTCATCGTACTCCTGCTTG3′; set 2, F2: 5′ GTAAAGACCTCTATGCCAACA3′, R2: 5′GGACTCATCGTACTCCTGCT3′) according to the following nested PCR protocol: first run: 30 cycles of 98°C for 10 s, 59°C for 15 s and 72°C for 5 s; second run: 35 cycles of 98°C for 10 s, 57°C for 15 s and 72°C for 5 s.

Real-time PCR was performed using Mx3000p QPCR system (Stratagene Corporation, La Jolla, CA, USA) with Brilliant II QPCR Master Mix (Agilent Technologies, Palo Alto, CA, USA). The target sequence was amplified in a total reaction volume of 10 µl containing 5 µl SYBR Green PCR Master Mix (2×), 0.5 µl cDNA (or cell contents not subjected to reverse transcribed) template and 0.5 µl forward/reverse primers (F: 5′CATCGCCAACTACTACGCTCTA 3′; R: 5′CGCCGTCATCCTCATCCT 3′) using the following PCR protocol: 40 cycles of 95°C for 30 s, 61°C for 30 s and 72°C for 20 s.

### Statistical analysis

Data were analyzed with the two-tailed unpaired Student's *t*-test using Sigmastat 3.5 software (Systat Software, Inc., San Jose, CA, USA), unless otherwise stated. Two-way ANOVA with *post-hoc* Holm-Sidak test was used to compare [Ca^2+^]_i_ between rat and ground squirrel neurons. All data are presented as mean ± s.e.m. The sample size used for statistical analysis was n. *P*<0.05 was considered to be statistically significant.

## Results

### Ground squirrel neurons were more tolerant to glutamate toxicity than rat neurons

**Figure 1:**
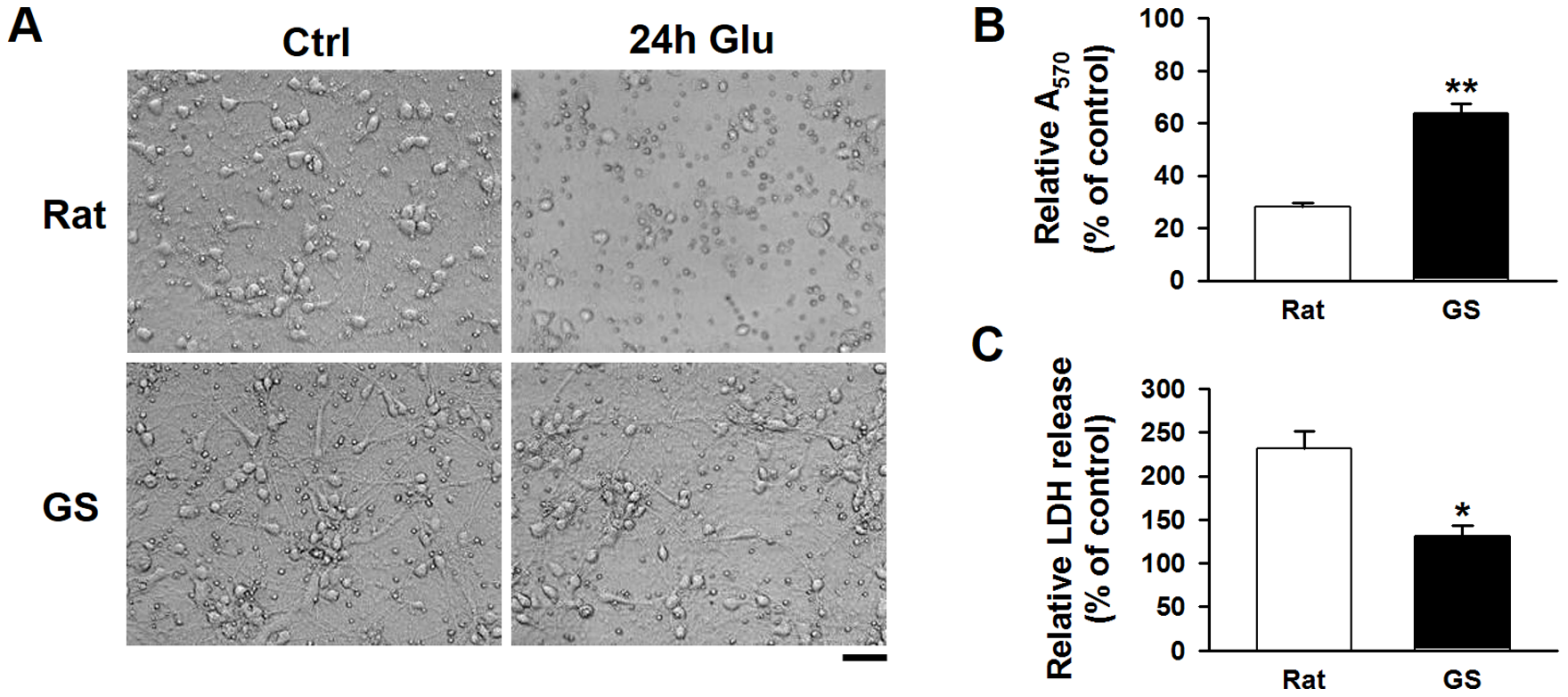
Ground squirrel primary cortical neurons were more tolerant to glutamate toxicity than rat neurons. After the primary cortical neurons were cultured for 9 days *in vitro*, glutamate (200 µM) was added to the culture for 24 hours. (A) Representative phase contrast images show ground squirrel and rat cultured cortical neurons. The images show that the morphology of the ground squirrel neurons is better maintained than that of the rat neurons. Scale bar, 50 µm. (B) MTT assay, A_570_ refers to the absorbance at 570 nm. n = 3 separate experiments, *P*<0.01. (C) LDH release assay. Rat: n = 3 separate experiments, GS: n = 2 separate experiments, *P*<0.05. The results demonstrate that ground squirrel neurons survived glutamate toxicity better than rat neurons. GS: ground squirrel.

Hibernators have been reported to be resistant to ischemic damage *in vivo* [2] and in brain slices [5], however, whether hibernators are resistant to ischemic insults or excitotoxicity (the key event in ischemia) at the cellular level is still undetermined. To test whether ground squirrel have an adaptive advantage against excitotoxicity over rat at the neuronal level, primary neurons were treated with glutamate to induce cell damage. Under basal conditions, no significant differences in cell viability were detected between rat and ground squirrel neurons, as determined by the MTT assay (Rat: 0.72±0.04; Ground squirrel: 0.77±0.32. *P* = 0.39, n = 3). However, following glutamate treatment for 24 hours, considerably less morphological defects were observed in ground squirrel neurons than in rat neurons (Fig. 1A). We further quantified the difference in cell viability using the MTT assay (Fig. 1B, Rat: 27.97±1.52%; Ground squirrel: 63.87±3.71%) and the LDH release assay (Fig. 1C, Rat: 231.34±20.37%; Ground squirrel: 131.29±11.50%). These results show the neuronal viability is much higher in ground squirrel, indicating that at the neuronal level ground squirrel is also more resistant to glutamate toxicity than rat.

Extensive activation of glutamate receptors can lead to overproduction of ROS and cause damage to lipids, proteins and DNA; therefore, ROS can be used as a marker of severe stress [23], [24]. To assess ROS changes in the cytoplasm, neurons were stained with DHE [25], [26]. Indeed, the DHE fluorescence intensity of rat neurons increased by more than 1.5 fold over 3 hours of glutamate treatment (Fig. S1A, 164±10.16%). However, no significant increase in the DHE fluorescence was detected in the ground squirrel neurons (Fig. S1A, 102±4.17%). This result suggests that ground squirrel neurons generate less ROS under glutamate toxicity. Furthermore, the ATP assay indicates that ATP level is higher in ground squirrel neurons (Fig. S1B, Rat: 32.93±3.47%; Ground squirrel: 62.51±3.82%). Mitochondrial dysfunction promotes cell death in various diseases [27]–[29]. To assess the mitochondrial membrane potential (Ψ_m_), we performed JC-1 staining [30]. The results showed that ground squirrel neurons maintained a stable Ψ_m_ after 3- or 24-hour glutamate treatment, whereas rat neurons displayed 37.62% or 41.31% reduction in the Ψ_m_ after the 3- or 24-hour glutamate treatment (Fig. S1C). Together, our results suggest that ground squirrel neurons have several adaptive advantages for resisting glutamate-induced cell death, including better maintenance of mitochondrial membrane potential and lower ROS production.

### Ground squirrel neurons maintained calcium homeostasis better than rat neurons

**Figure 2:**
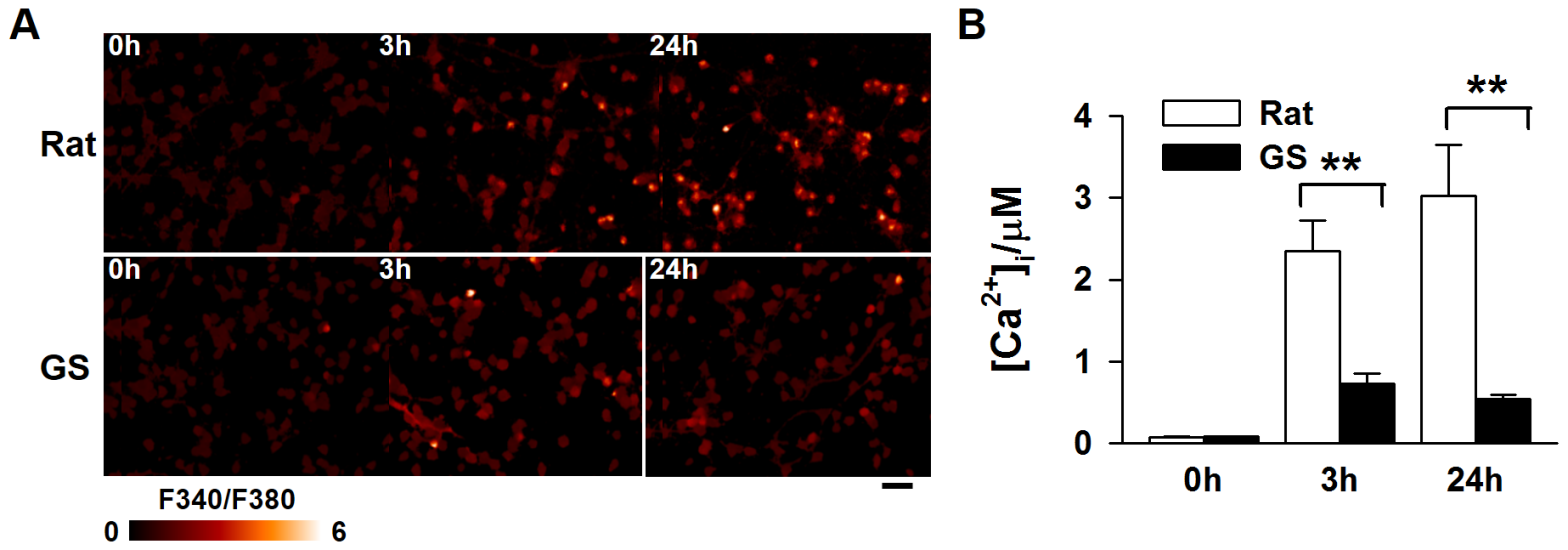
The [Ca^2+^]_i_ was much lower in ground squirrel neurons 3 and 24 hours after glutamate exposure. (A) Representative fluorescence ratio images of fura-2. Scale bar: 50 µm. (B) Statistical results show that the relative intracellular calcium concentration ([Ca^2+^]_i_) in neurons after glutamate exposure is increased in both ground squirrel and rat neurons. However, the level of the [Ca^2+^]_i_ increase was much lower in the ground squirrel neurons than in the rat neurons. n = 174–180 neurons, from 3 separate experiments. Two-way ANOVA test with *Post hoc* Holm-Sidak comparison, *P*<0.01. Fura-2 AM was used as a calcium indicator.

Glutamate-induced mitochondrial damage and cell death are primarily caused by calcium overload [31]–[33], the higher viability of ground squirrel neurons under glutamate treatment described above thus may result from the better regulation of calcium homeostasis in these cells. To test whether the [Ca^2+^]_i_ in ground squirrel neurons is different from that in rat neurons during the glutamate treatment, we measured [Ca^2+^]_i_ with fura-2, a ratiometric calcium indicator. At the resting state, the [Ca^2+^]_i_ of rat and ground squirrel neurons displayed a similar basal level (Fig. 2). However, the increase in [Ca^2+^]_i_ was notably less in ground squirrel neurons than in rat neurons after 3-hour or 24-hour glutamate treatment (Fig. 2, 0 hours: Rat: 75.91±8.29 nM; Ground squirrel: 79.96±6.22 nM; 3 hours: Rat: 2.35±0.38 µM; Ground squirrel: 0.72±0.13 µM; 24 hours: Rat: 3.03±0.62 µM; Ground squirrel: 0.54±0.05 µM). This result indicates that ground squirrel neurons can better maintain calcium homeostasis than rat neurons during glutamate exposure.

### Glutamate and NMDA induced [Ca^2+^]_i_ elevations were similar in ground squirrel and rat neurons

**Figure 3:**
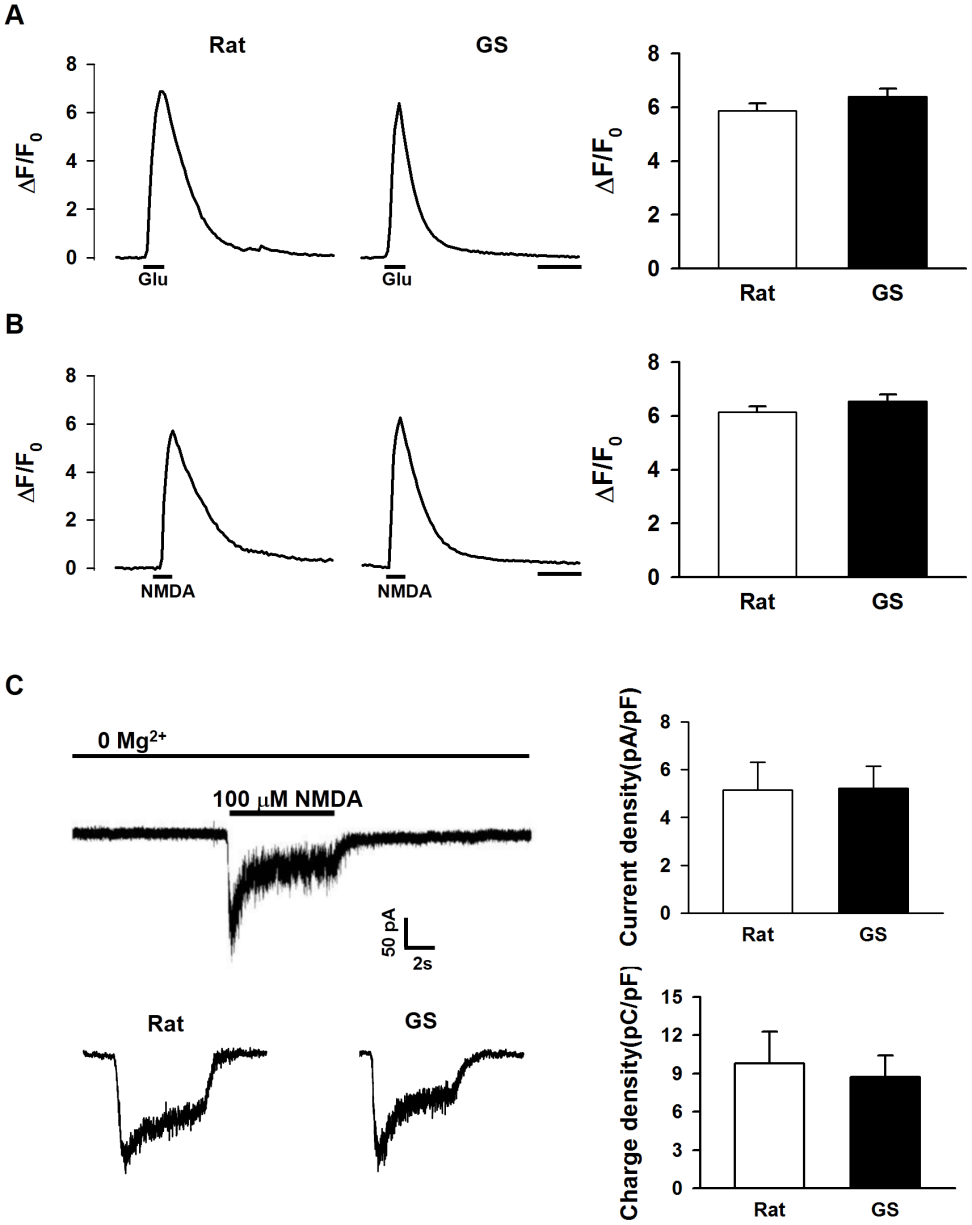
A similar [Ca^2+^]_i_ elevation in rat and ground squirrel neurons was induced by glutamate and NMDA. (A) Glutamate (Glu, 200 µM) induced [Ca^2+^]_i_ changes in ground squirrel and rat neurons. n = 58–65 neurons, from 3 separate experiments. Scale bar, 10 s. (B) NMDA (100 µM) induced [Ca^2+^]_i_ elevation in both types of neurons. Scale bar, 10 s. The results show that the elevation in [Ca^2+^]_i_ induced by glutamate or NMDA is similar in the two types of neurons. n = 55–62 neurons, from 3 separate experiments. (C) The neuronal response to NMDA perfusion was measured by whole-cell patch clamp. The results show that no significant differences exist in current density or charge density between ground squirrel and rat neurons. Eighteen rat neurons (from 5 separate experiments) and 26 ground squirrel neurons (from 7 separate experiments) were examined and used for statistical analysis. Fluo-4 AM was used in A and B as a calcium indicator.

[Ca^2+^]_i_ is determined by the combined effects of calcium influx/release into and the removal from the cytosol. Technically speaking, calcium overload can be achieved by over-activation of calcium influx/release and/or decrease of calcium removal. Another calcium indicator with lower affinity (fluo-4) than fura-2 was used to measure the [Ca^2+^]_i_ elevation. We found no significant difference in the amplitude of the [Ca^2+^]_i_ elevation between ground squirrel and rat neurons upon glutamate perfusion (Fig. 3A, Rat: 5.87±0.27; Ground squirrel: 6.40±0.28). NMDA receptor is the main glutamate receptor mediating Ca^2+^ influx and [Ca^2+^]_i_ elevation. The amplitude of the [Ca^2+^]_i_ elevation induced by NMDA perfusion was thus measured and showed no difference between the two types of neurons either (Fig. 3B, Rat: 6.14±0.22; Ground squirrel: 6.54±0.27). We also measured NMDA receptor-mediated currents by whole-cell patch clamp recording. In coincidence with previous results, there were no differences in current density (Fig. 3C, Rat: 5.15±1.16 pA/pF; Ground squirrel: 5.21±0.92 pA/pF) or charge transfer density (Fig. 3C, Rat: 9.80±2.48 pC/pF; Ground squirrel: 8.74±1.66 pC/pF) between ground squirrel and rat neurons. Together, these results suggest that the influx of calcium and [Ca^2+^]_i_ elevation in response to glutamate stimulation is similar in ground squirrel and rat neurons.

### Calcium removal mediated by NCX was faster in ground squirrel neurons than in rat neurons

**Figure 4:**
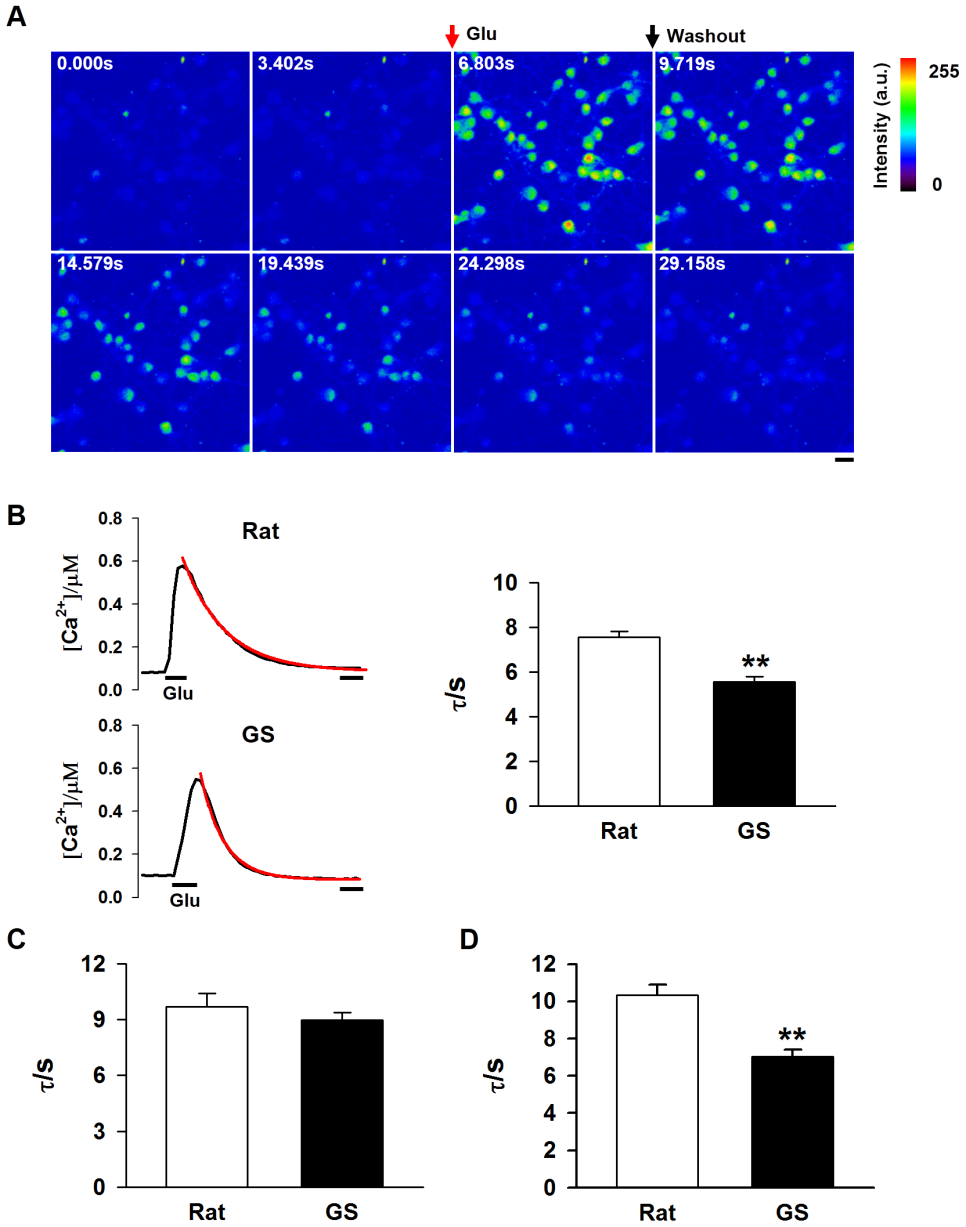
The velocity of calcium removal was faster in ground squirrel neurons than in rat neurons. (A) A series of image frames of fluo-4 AM loaded neurons acquired by confocal microscopy. Glutamate (Glu) was applied between the second and the third frame (indicated by the red arrow). Then, glutamate was washed out approximately 2 s later (indicated by the black arrow). Scale bar, 20 µm. (B) Left: a typical example of [Ca^2+^]_i_ dynamics in response to glutamate administration (the black line). The decay phase is fit with a single exponential function (red line) to estimate the time constant (τ). Right: statistical results suggest that the calcium removal rate is markedly faster in ground squirrel neurons than rat neurons. n = 76 to 131 neurons, from 3 separate experiments, *P*<0.01. Scale bar, 5 s. (C) Effect of NCX blockade on the [Ca^2+^]_i_ decay rate. The result shows that the reduced τ value in ground squirrel neurons is abolished by administration of CB-DMB, a specific NCX blocker. n = 73 to 96 neurons, from 5 separate experiments. (D) Effect on the [Ca^2+^]_i_ decay rate of the inhibition of SERCA. The results show that the reduced calcium removal rate is similar in rat and ground squirrel neurons in response to TG, a SERCA blocker. n = 56 to 70 neurons, from 4 (rat) and 3 (GS) separate experiments.

The rate of calcium removal in the cytoplasm can be assessed by the time constant (τ), which is estimated by fitting the [Ca^2+^]_i_ decay phase with a single exponential function [34]. Glutamate perfusion induced a calcium transient in ground squirrel and rat neurons. As illustrated in Fig. 4A and 4B, the decay τ of ground squirrel neurons was lower, indicating a faster calcium removal rate than in rat neurons after glutamate washout (Fig. 4B, τ_rat_ = 7.56±0.27 s; τ_ground squirrel_ = 5.56±0.22 s).

The major calcium removal pathways are through NCX on the plasma membrane and SERCA on the ER. First, we examined the effect of blocking NCX by CB-DMB, a pan- specific inhibitor of the NCXs in excitable cells [35], [36]. We found that CB-DMB could significantly prolong the decay time in both ground squirrel and rat neurons, resulting in comparable values of τ in the two species (Fig. 4C, τ_rat_ = 9.69±0.74 s; τ_ground squirrel_ = 8.98±0.39 s). The blockade of NCX was sufficient to eliminate the difference of τ between ground squirrel and rat neurons, indicating that the activity of NCXs is likely higher in ground squirrel neurons than in rat neurons. However, TG, a specific SERCA inhibitor, equally increased τ in both ground squirrel and rat neurons (Fig. 4D, Rat-TG: τ = 10.33±0.55 s; Ground squirrel-TG: τ = 7.38±0.37 s). The decrease of calcium removal rate was similar in ground squirrel and rat neurons (Rat: 0.028±0.011 s^−1^; Ground squirrel: 0.015±0.0098 s^−1^. *P* = 0.36, n = 3), suggesting that ground squirrel and rat neurons have similar SERCA activity.

### Expression of ground squirrel NCX2 in rat neurons increased neuron survival against glutamate toxicity

**Figure 5:**
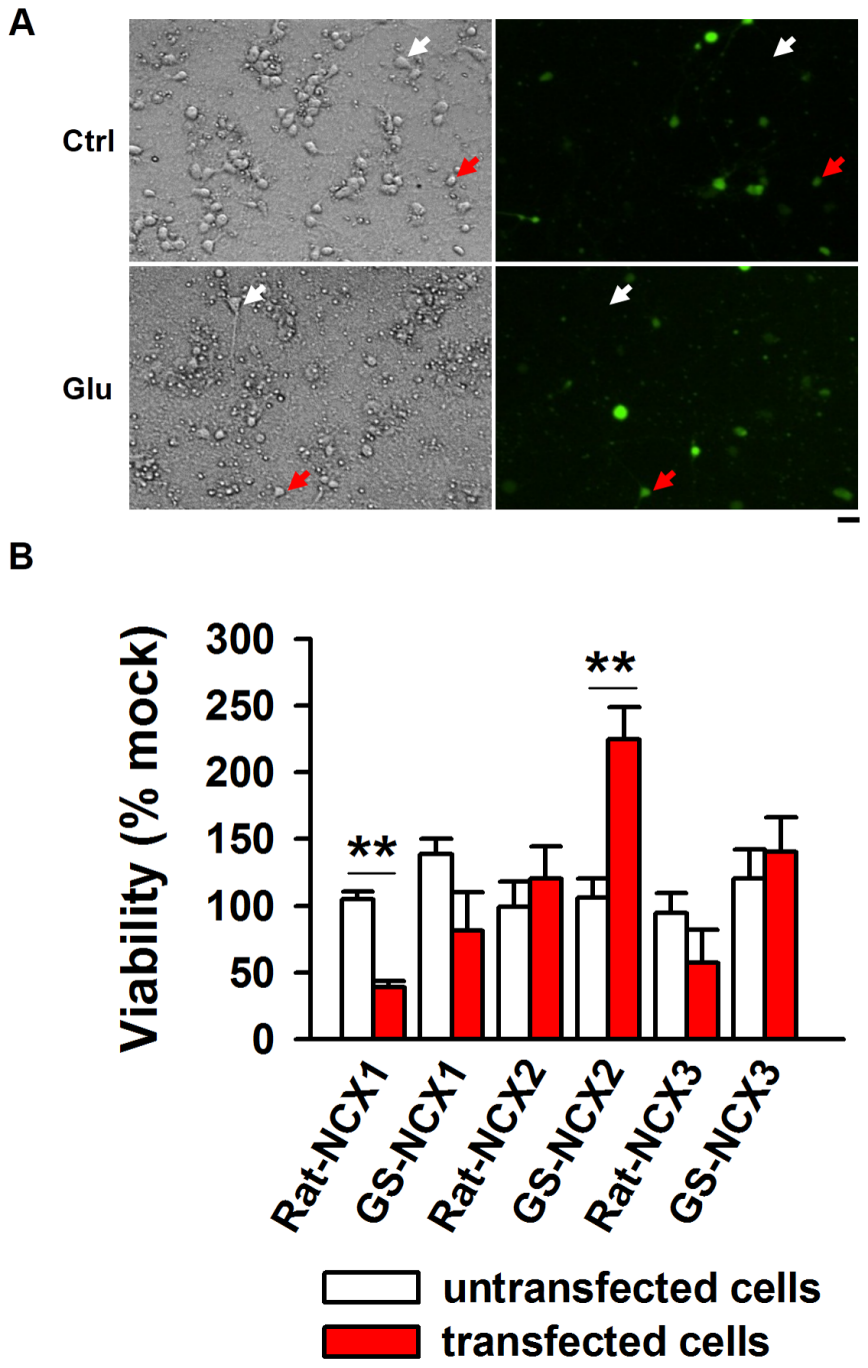
Ground squirrel NCX2 expression in rat primary neurons increased the viability of neurons exposed to glutamate. (A) Expression of ground squirrel NCX2 in rat primary neurons. Neurons transfected with ground squirrel *NCX2* were labeled by EGFP, and neuronal survival was determined 6 hours after glutamate treatment. Scale bar, 25 µm. The results show that neurons transfected with ground squirrel *NCX2* (EGFP positive cells identified by red arrows) are less susceptible to glutamate injury than were untransfected neurons (identified by white arrows). In addition, the effect of overexpressing NCX1, NCX2 and NCX3 from both rat and ground squirrel neurons were tested. The results are shown in the bar graph (B). The data suggest that the expression of ground squirrel NCX2 promotes survival under glutamate toxicity conditions, whereas overexpression of rat NCX1 enhances cell death. In contrast, overexpression of other rat and ground squirrel NCXs has no effect. n = 5 separate experiments, *P*<0.01.

The rat genome contains three *NCX* genes, so does the ground squirrel genome. To test which *NCX* gene plays a protective role against glutamate toxicity, we transfected the cDNA of these *NCX* genes individually into rat neurons using EGFP as an indicator of the NCX expression (the transfection rate is approximately 10%). The neuronal survival after glutamate treatment was quantified by counting live cells [37]. We observed that the survival rate was similar among untransfected neurons in all groups (Fig. 5A, B). However, neurons overexpressing ground squirrel *NCX2* displayed resistance to glutamate toxicity, and the viability of these cells was 224.59% of the control group (Fig. 5B). The mRNA expression of ground squirrel *NCX2* was confirmed using single-cell PCR method (Fig. S2A and B). We further evaluated the calcium removal rate with the red-shift calcium indicator rhod-2 in rat neurons transfected with the empty vector or the vector containing ground squirrel *NCX2*. The calcium removal rate was similar in untransfected and empty vector-transfected neurons (untransfected neurons: 10.44±0.54 s; empty vector-transfected neurons: 10.26±0.89 s. *P* = 0.86, n = 5); however, calcium removal was markedly faster in the neurons expressing ground squirrel NCX2 (Fig. S3, Mock: 10.97±1.24 s; Ground squirrel NCX2: 6.22±0.50 s). Together, these results suggest that the expression of ground squirrel NCX2 increases rat neuron survival against glutamate toxicity.

## Discussion

The present study unveils ground squirrel is more tolerant to glutamate toxicity than rat also at the neuronal level, based on a lower [Ca^2+^]_i_ level during glutamate exposure. This maintenance of calcium homeostasis could be explained by a faster calcium removal rate resulted from higher activity of NCX in ground squirrel neurons. Expression of ground squirrel NCX2 in rat neurons reduces rat neuron death induced by glutamate excitotoxicity. Our findings might reveal an adaptive mechanism protecting against ischemic injury during hibernation, at the neuronal level.

It is well accepted that hibernators survive ischemia much better than nonhibernators, as determined *in vitro* [4], [17] and *in vivo* [2]. However, much remains to be learned about the adaptive mechanisms involved. In the present study, we focused on intracellular calcium homeostasis during glutamate exposure in primary cultured cortical neurons of rats and ground squirrels. We demonstrated that ground squirrel neurons were more resistant to glutamate excitotoxicity than rat neurons (Fig. 1). Mechanistically, the protective effect was a result of maintaining low [Ca^2+^]_i_ in ground squirrel neurons during glutamate exposure (Fig. 2). A previous study has demonstrated that glutamate could induced a lower [Ca^2+^]_i_ elevation in the hippocampal slices of hibernating and interbout euthermic AGS than in rats [38]. In our study, we showed that the amplitude of the [Ca^2+^]_i_ elevation elicited by glutamate administration was similar in rat and ground squirrel primary neurons (Fig. 3A). In addition to the amplitude of calcium elevation [39], the source of calcium [40] is another determinant of glutamate toxicity. The NMDA receptor-mediated calcium influx has a particular propensity to cause neuronal injury [41]. However, the role of the NMDA receptor in mediating neuronal death in hibernators and non-hibernators remains controversial. Some studies proposed that the NMDA receptor played a key role, because the expression of the NR1 subunit of the NMDA receptor [38] and NMDA receptor-mediated neuronal death [4] were lower in hibernating and interbout euthermic AGS than in rats. However, no difference in the NR1 expression between rats and euthermic hamsters, while euthermic hamsters were more tolerant to OGD (oxygen and glucose deprivation) than rats [42] was also reported. Our data suggest that the NMDA receptor-mediated currents between rat and ground squirrel neurons are identical (Fig. 3B, C), indicating a similar role of the NMDA receptor in mediating glutamate neurotoxicity in the primary neurons of these two species.

During sustained glutamate exposure, high-capacity calcium removal apparatuses are required to remove the overloaded calcium elicited by glutamate. In contrast to PMCA clustering at the active zone, NCX localizes at the neuronal soma. Distinct from PMCA with a high affinity for Ca^2+^ (*K*_d_≈100 nM) and a low turnover rate (30–250 s^−1^), NCX has a lower affinity for Ca^2+^ (*K*_d_≈1000 nM) and a much higher turnover rate (2,000–5,000 s^−1^) [43]. These properties allow NCX to quickly remove calcium upon calcium overload. We found that a higher NCX activity in ground squirrel neurons (Fig. 4) facilitated calcium extrusion. NCX is a bi-directional membrane ion transporter and can shift to a reverse mode (injury promoting) under certain conditions [44], [45]. Furthermore, NCX isoforms could be cleaved by diverse proteases [46], [47]. For instance, NCX1 and NCX3 were degraded in focal brain ischemia, and NCX3 was cleaved in glutamate excitotoxicity in cerebellar granule neurons; in contrast, NCX2 was not cleaved during focal brain ischemia or glutamate exposure [48]. The role of NCX1 in ischemia injury is highly debated. Overexpression of cardiac NCX1 was reported to exacerbate ischemia/reperfusion injury in male transgenic mice [49], while knockdown of NCX1 in cortical neurons decreased cell death by ∼30% in response to OGD/reperfusion injury [50], both of which were consistent with our observation that overexpression NCX1 in rat primary neurons increased cell death (Fig. 5A and B). It is still unknown what causes the difference between NCX1 and the other two isoforms, one possibility is that the equilibrium potential for the three isoforms might be different.

Furthermore, our data suggest that the expression of ground squirrel NCX2, rather than other NCXs, promotes neuronal survival from glutamate toxicity (Fig. 5A and B). We did sequence analysis to explore the possible causes. After multiple sequence alignment analysis of NCX2, we next performed protein phosphorylation sites prediction with DISPHOS and Netphos 2.0. As a result, a predicted phosphorylation site (DISPHOS: 0.725; Netphos 2.0: 0.989) was uncovered in ground squirrel and another hibernator (thirteen-lined ground squirrel, *Ictidomys tridecemlineatus*) without shown in rat or human. Aligned with dog (*Canis lupus familiaris*) NCX1 3D structure (NCX2 3D structure are unknown), this phosphorylation site locates near the beginning of calcium binding domain 1 (CBD1), which may be involved in its activity regulation. We are working on this possibility in collaboration with another group. Bano et al. [48] reported that overexpression of rat NCX2 protected cerebellar granule neurons from glutamate toxicity, which we did not observe. A more severe damage in our model (200 µM glutamate for 6 hours in our system vs. 150 µM for 2.5 hours in their system) and a different cell type (the cortical neurons in our system vs. cerebellar granule neurons in their experiment) we used might account for this inconsistency.

Actually, in addition to the failure of intracellular calcium homeostasis, the increase of ROS production, the decrease of mitochondrial membrane potential and ATP level, as well as the crosstalk among them also contributed to glutamate-induced neuronal injury [51], [52]. We observed less ROS production (Fig. S1A), higher ATP level (Fig. S1B) and better maintained mitochondrial membrane potential (Fig. S1C) in ground squirrel neurons than in rat neurons under glutamate treatment. We also found a lower ROS level and comparable mitochondrial membrane potential in rat primary neurons transfected with ground squirrel *NCX2* (Fig. S4A and B), indicating increased ROS level, rather than loss of mitochondrial membrane potential, was a consequence of limited calcium clearance. Furthermore, the decreased ROS level in rat primary neurons expressed ground squirrel NCX2 was also consistent with our previous observation that ROS formation in ground squirrel neurons was less than in rat neurons (Fig. S4A).

Glutamate excitotoxicity widely occurs in neurodegenerative disorders, such as seizures, ischemia and traumatic brain injury. To develop strategies preventing neuronal cells from death, most studies have focused on reducing calcium entry, such as inhibition of the NMDA receptor. Here, our research indicates that increased calcium extrusion can also protect neurons from glutamate toxicity. Our research may promote further investigation into the function of ground squirrel NCX2 and provide novel targets for treating excitotoxicity-mediated neurological disorders.

## Supporting Information

Figure S1 **Compared with rat neurons, ground squirrel neurons maintained lower reactive oxygen species (ROS) production (A. DHE was used as a ROS indicator. n = 146 to 153 neurons, from 3 separate experiments,** ***P*** **<0.05), higher ATP level (B. Rat: n = 3 separate experiments; Ground squirrel: n = 5 separate experiments,** ***P*** **<0.01) and more stable mitochondrial membrane potential (Ψ_m_) (C. JC-1 was used as Ψ_m_ indicator.** Rat: n = 3 separate experiments; Ground squirrel: n = 5 separate experiments, *P*<0.05) under glutamate treatment. GS: ground squirrel.
(TIF)

Figure S2 **The expression of ground squirrel** ***NCX2*** **mRNA was detected in transfected rat neurons.** (A) Ground squirrel (GS) *NCX2* specific band was detected only in reverse-transcribed preparations from transfected neurons. 0: control (no cells); B: nontransfected neurons; E: mock transfected neurons; G: GS *NCX2* transfected neurons. n = 3 separate experiments. (B) Both the GS *NCX2* plasmid and the mRNA transcribed from GS *NCX2* could serve as templates for the PCR amplification. Their roles were determined with Q-PCR. cDNA: mRNA of GS *NCX2* as template; P: plasmid GS-NCX2 as template. n = 7–9 neurons, from 3 separate experiments, *P*<0.01. GS: ground squirrel.
(TIF)

Figure S3 **Expression of ground squirrel NCX2 in rat primary neurons reduced the τ value of calcium removal.** Neurons were loaded with 10 µM rhod-2 AM for 10 min at 37°C. n = 24–28 neuron, from 5 separate experiments, *P*<0.01.
(TIF)

Figure S4 **Ground squirrel NCX2 expression in rat primary neurons decreased ROS, and had no effect on mitochondrial membrane potential (Ψ_m_).** (A) Left: ROS level of rat primary neurons was significantly higher than that in ground squirrel neurons. Rat: n = 153 neurons, from 4 separate experiments; GS: n = 147 neurons, from 3 separate experiments, *P*<0.01. Right: expression of GS NCX2 in rat primary neurons lowered ROS level. n = 20 to 55 neurons, from 3 separate experiments, *P*<0.01. (B) Expression of GS NCX2 in rat primary neurons did not change Ψ_m_. n = 109, 32, 120, 44 neurons respectively, from 3 separate experiments. Two-way ANOVA test. GS: ground squirrel.
(TIF)
