# Supplementary figures and images for: Increased Na+/Ca2+ Exchanger Activity Promotes Resistance to Excitotoxicity in Cortical Neurons of the Ground Squirrel (a Hibernator)
Source: PLoS One. 2014 Nov 21;9(11):e113594. doi: 10.1371/journal.pone.0113594 (PMC4240599; doi:10.1371/journal.pone.0113594)

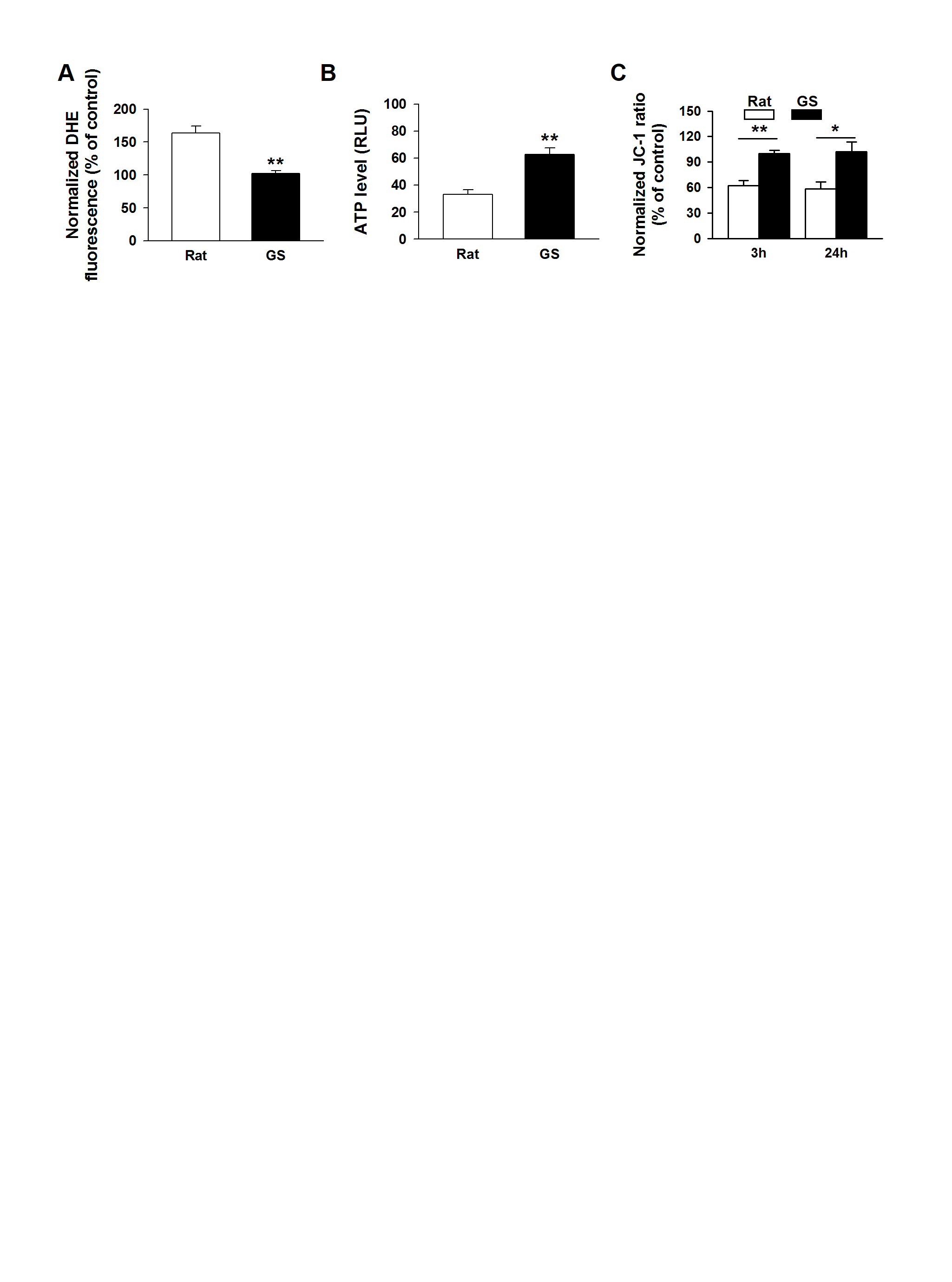

Supplement: Figure S1 — Compared with rat neurons, ground squirrel neurons maintained lower reactive oxygen species (ROS) production (A. DHE was used as a ROS indicator. n = 146 to 153 neurons, from 3 separate experiments, P <0.05), higher ATP level (B. Rat: n = 3 separate experiments; Ground squirrel: n = 5 separate experiments, P <0.01) and more stable mitochondrial membrane potential (Ψm) (C. JC-1 was used as Ψm indicator. Rat: n = 3 separate experiments; Ground squirrel: n = 5 separate experiments, P<0.05) under glutamate treatment. GS: ground squirrel. (TIF) [file pone.0113594.s001.tif]

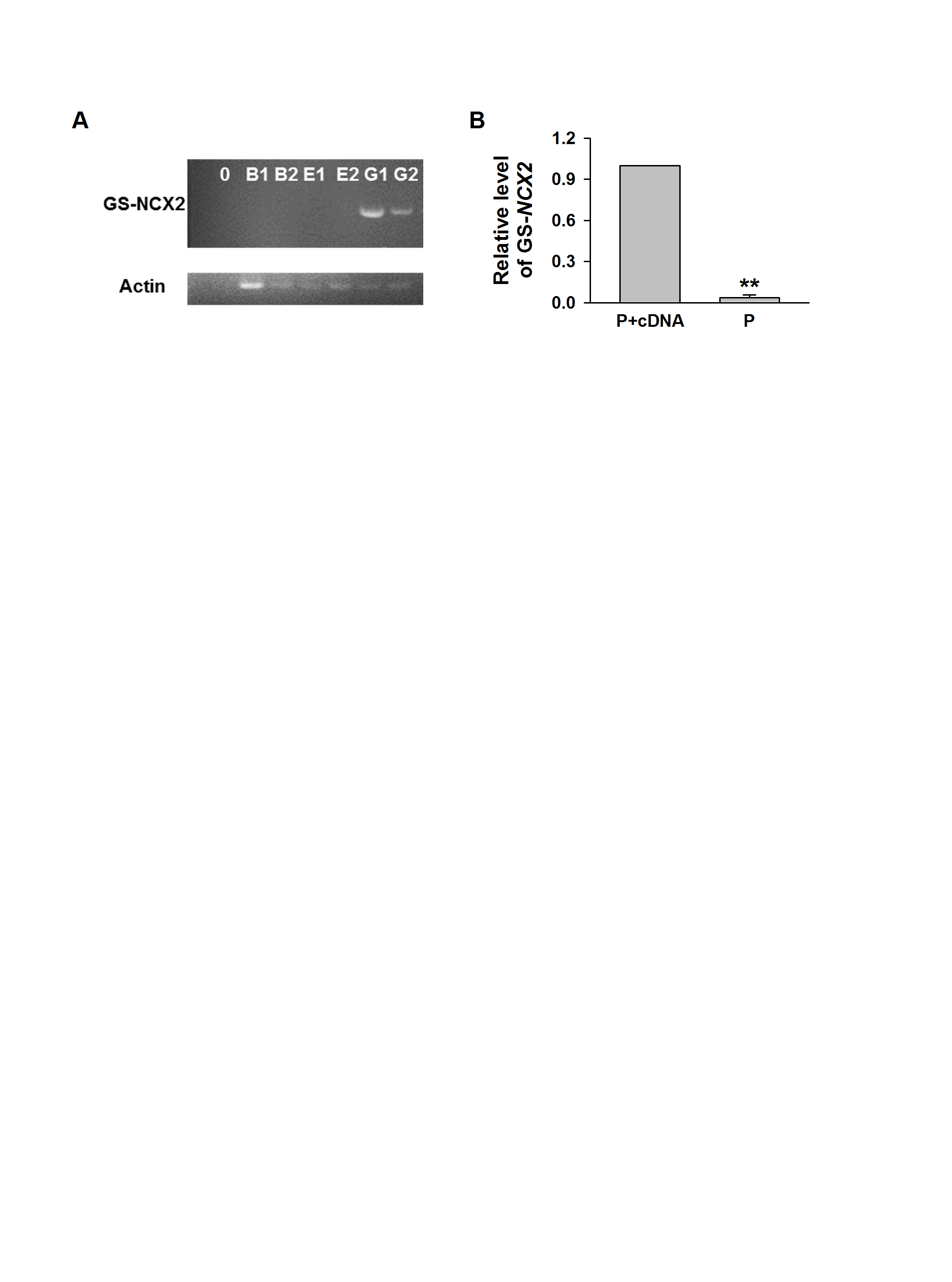

Supplement: Figure S2 — The expression of ground squirrel NCX2 mRNA was detected in transfected rat neurons. (A) Ground squirrel (GS) NCX2 specific band was detected only in reverse-transcribed preparations from transfected neurons. 0: control (no cells); B: nontransfected neurons; E: mock transfected neurons; G: GS NCX2 transfected neurons. n = 3 separate experiments. (B) Both the GS NCX2 plasmid and the mRNA transcribed from GS NCX2 could serve as templates for the PCR amplification. Their roles were determined with Q-PCR. cDNA: mRNA of GS NCX2 as template; P: plasmid GS-NCX2 as template. n = 7–9 neurons, from 3 separate experiments, P<0.01. GS: ground squirrel. (TIF) [file pone.0113594.s002.tif]

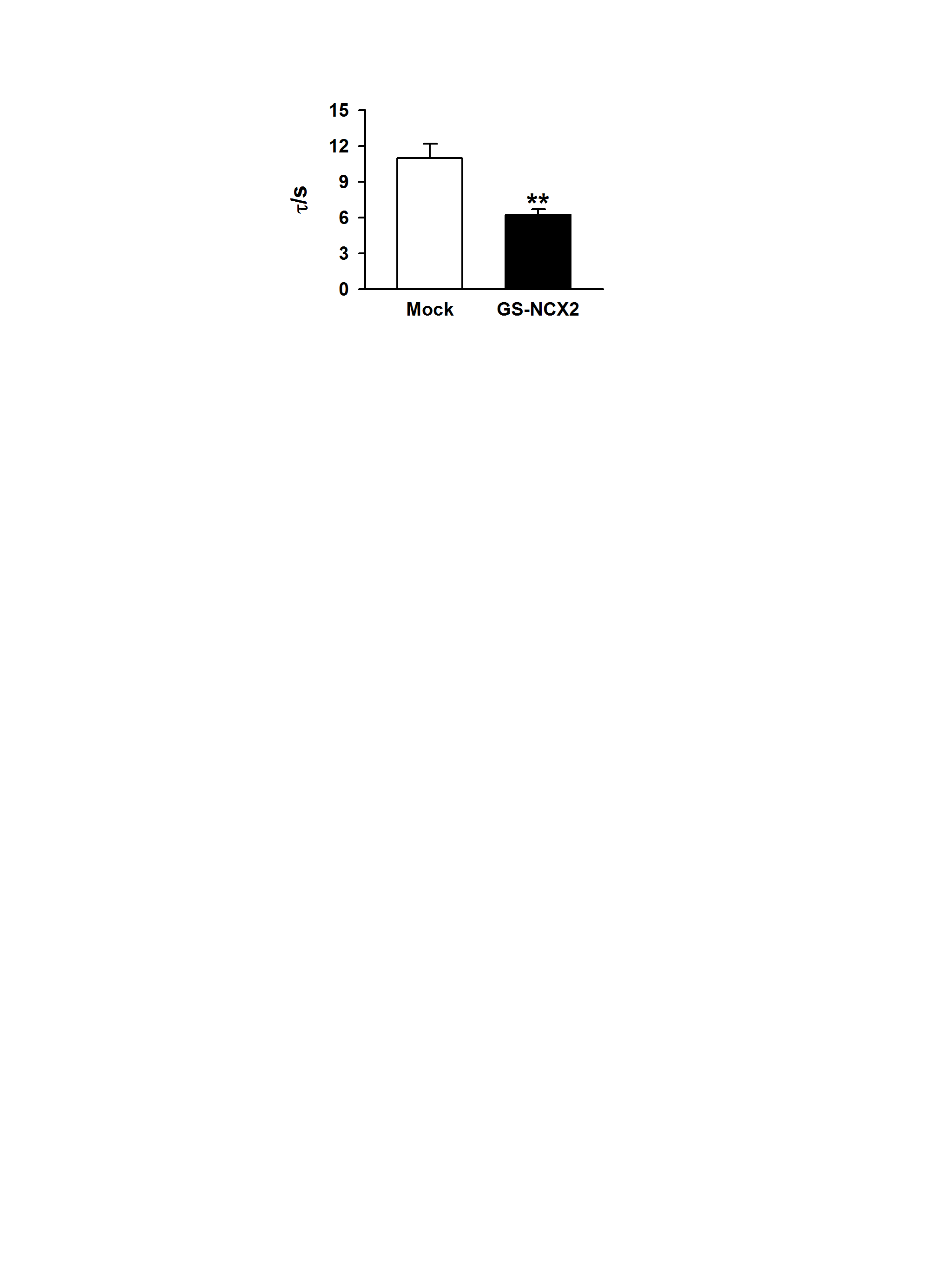

Supplement: Figure S3 — Expression of ground squirrel NCX2 in rat primary neurons reduced the τ value of calcium removal. Neurons were loaded with 10 µM rhod-2 AM for 10 min at 37°C. n = 24–28 neuron, from 5 separate experiments, P<0.01. (TIF) [file pone.0113594.s003.tif]

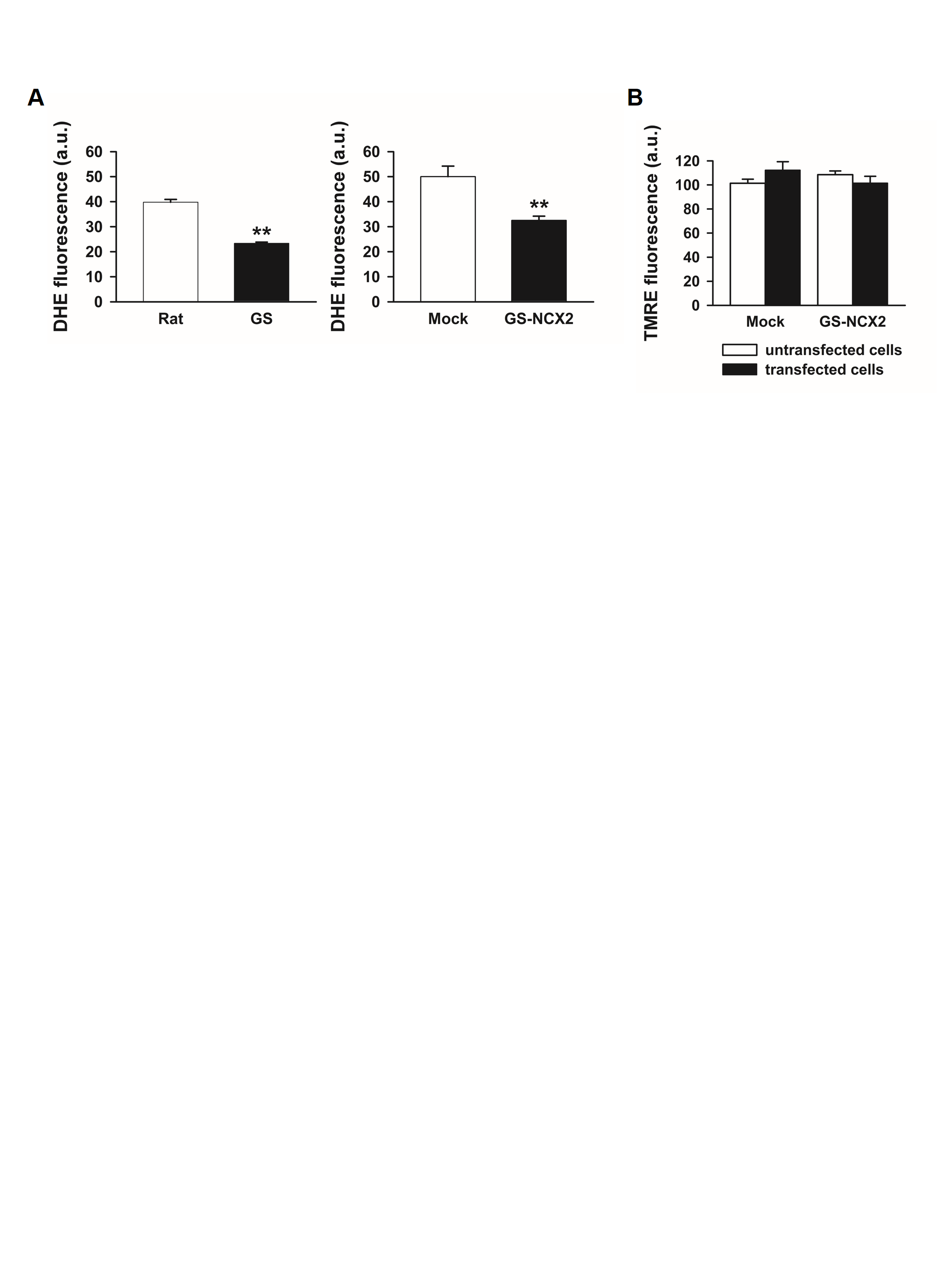

Supplement: Figure S4 — Ground squirrel NCX2 expression in rat primary neurons decreased ROS, and had no effect on mitochondrial membrane potential (Ψm). (A) Left: ROS level of rat primary neurons was significantly higher than that in ground squirrel neurons. Rat: n = 153 neurons, from 4 separate experiments; GS: n = 147 neurons, from 3 separate experiments, P<0.01. Right: expression of GS NCX2 in rat primary neurons lowered ROS level. n = 20 to 55 neurons, from 3 separate experiments, P<0.01. (B) Expression of GS NCX2 in rat primary neurons did not change Ψm. n = 109, 32, 120, 44 neurons respectively, from 3 separate experiments. Two-way ANOVA test. GS: ground squirrel. (TIF) [file pone.0113594.s004.tif]
